# Peer-assisted learning - an antidote for spoonfeeding? Reflections on peer-assisted learning activites in a veterinary curriculum

**DOI:** 10.15694/mep.2017.000159

**Published:** 2017-09-15

**Authors:** Alison Reid, Rosie MacDiarmid, Emma Ormandy, Karen Noble, Fay Penrose

**Affiliations:** 1 University of Liverpool

**Keywords:** Peer-assisted learning, Collaborative learning, Learning skills

## Abstract

This article was migrated. The article was marked as recommended.

Peer-assisted learning (PAL) is a potentially valuable teaching tool for students on veterinary and medical curricula, helping them to develop crucial learning, teaching and meta-adaptive skills ( Lizzio & Wilson, 2004) which will serve them during their undergraduate studies and throughout their future careers. This reflective article describes experiences of PAL activities on a UK veterinary degree course, and discusses potential reasons for success and failure of such activities. Advice is given for anyone planning to implement, or reviewing their own experiences of PAL.

## Introduction

Peer-assisted learning (PAL) has been described as active discussion and cooperative learning between peers ( Capstick, 2003). Any time a learner, in any sphere, discusses their learning with someone else in the same situation, this can arguably be described as PAL: if a friend at nursery school helps you learn to tie your shoelaces, this is a form of PAL. This is, therefore, clearly not a new phenomenon. In the higher education (HE) context, PAL may be formal, as part of the curriculum, or informal, occurring *ad hoc* among students ( Sevenhuysen *et al*., 2014). PAL has been in explicit formal use in HE since the 1990s ( Green, 2011), and its potential usefulness in veterinary and medical education is becoming increasingly clear.

PAL in higher education can be further defined as horizontal, with classmates working together within a cohort, or vertical, with more advanced students helping less experienced students to learn ( Black & Mackenzie, 2008). PAL activities may be student- or teacher-mediated ( Havnes, 2008). These basic factors, along with other considerations, may have a considerable impact on student engagement with and, hence, the success of PAL within a curriculum, as discussed below.

## Context

Veterinary curricula have much in common with their human medical counterparts, in terms of subject matter studied, workload undertaken and the types of undergraduates these courses attract. Students experience a wide variety of learning activities and study a vast array of subject matter, meaning the curriculum is dense and requires dedication and a strong work ethic from students.

In the UK, veterinary and medical undergraduate courses are typically five years’ long. Most veterinary curricula are structured with dense, direct teaching during the first 3-4 years followed by an extended period of clinical rotations during the final 12-18 months. The BVSc curriculum at the University of Liverpool has around 30 timetabled hours per week during the first 4 years, comprising a wide variety of learning activities, although around 50% is still lecture-based teaching. The BVSc, in common with most veterinary curricula in the UK, leads to a professional qualification which is accredited by the Royal College of Veterinary Surgeons. UK veterinary degrees are therefore, by necessity, somewhat exam-centred in order to ensure students meet the required standard upon graduation.

Kyndt *et al.* (2011), state that any professional graduate requires critical thinking skills, problem solving ability, the capacity to reflect and to be capable of self-management. These skills are particularly crucial for graduates entering the rapidly-changing medical (and, by extrapolation, veterinary) professions ( Towle & Cottrell, 1996). The RCVS sets out the skills required of a veterinarian at the point of graduation; the day one competences (RCVS, 2014). Included in this document are several competences which emphasise the need for strong learning skills in veterinary graduates, shown in Table One.

**Table One:** RCVS Day 1 Competences - learning skills

Number	Competence
11	Use professional capabilities to **contribute to the advancement of veterinary knowledge**, in order to improve the quality of animal care and public health
12	Demonstrate ability to **cope with incomplete information**, deal with contingencies and adapt to change
14	Demonstrate a **commitment to learning and professional development, both personal and as a member of a profession actively engaged in work-based learning**. This includes recording and reflecting on professional experience and taking measures to improve performance and competence.
15	Take part in **self-audit and peer-group review** processes in order to improve performance

Students enrolled on the BVSc are typical of veterinary and medical undergraduates everywhere in the UK. They tend to be high achieving and motivated, but also highly strategic, focussing their attentions on methods and information they think likely to lead to success in their exams ( Bennett *et al.*, 2015; Sykes *et al.*, 2011), rather than considering what they need for long term knowledge and learning skills. Our intake is around 70% school leavers, with the remainder being a mixture of graduates, mature students returning to study and access students.

The atmosphere in UK HE has shifted in the last few years, with greatly increased tuition fees for home students. Students are beginning to be viewed (and perhaps view themselves) as consumers and the temptation is to cater to their demands to increase satisfaction, regardless of the educational implications of this. Informal feedback from some students on the BVSc also highlights that this increased cost of study has a negative impact on student wellbeing; some students, already very driven and highly self-critical, feel excessive pressure to succeed since they and their families are investing such a large amount in their education. This may serve to further feed the exam-centric view many veterinary students take to their work. For educators on this type of course, a major challenge is getting past that exam-focus and enabling students to develop the lifelong learning skills so crucial to success beyond university.

Veterinary undergraduates have an additional factor to manage, over that experienced by their medical colleagues; there is no compulsory post-registration training phase for veterinary students. This means that veterinary graduates must have fully developed skills, including learning skills, to enable them to adapt to their chosen field and work autonomously from day one.

It is not usual to assess learning skills in of themselves, and yet these are clearly required for success as a veterinarian. The onus is on us as teachers on veterinary, and medical, curricula to ensure that development of life-long learning skills is embedded into the explicit and implicit curriculum in such a way that any student advancing through the course must gain these skills by the time they graduate.

## Where does peer-assisted learning come in?

Educators employ various techniques to endeavour to address the issues highlighted above. Alternative teaching methods such as problem-based learning, the “flipped classroom” approach, small group teaching, case-based seminars and reflective portfolios are all efforts to supplement (or indeed replace) didactic teaching with more active learning experiences.

Student-led activities such as PAL can be included in any of these approaches, and can stand alone as learning activities in their own right. Several authors, including Wilson and Fowler (2005) have suggested that student-led learning activities can promote deep learning of subject matter, and it is clear that the very act of giving students ownership of their own learning will foster development of the desirable learning skills so necessary in veterinary and medical professionals. Lizzio and Wilson (2004), demonstrated that active involvement in and responsibility for the learning process deepen learning approaches - it is this active involvement that is key. This presents educators with the greatest challenge; we can lead a student to class, but we cannot make him learn.

In courses like the BVSc, students spend a great deal of time together and usually form a close-knit social community. This gives wide scope for informal and student-mediated PAL, if this approach to learning can be fostered in our students.

PAL provides students with a safe place; a less intimidating space to ask and answer questions without fear of showing a lack of knowledge to their teacher (Baillie *et al*, 2009; Glynn *et al*, 2006). This increases active learning; a clearly desirable feature. In vertical PAL, there are benefits for the peer tutor and the peer tutee, both in terms of knowledge gain and learning skill development ( Topping & Francis, 2005; Williams & Reddy, 2016). Development of a peer-learning ethos contributes to positive aspects of the “hidden curriculum” so often discussed, encouraging self-directed learning to become an accepted norm and a behaviour to be modelled in the future ( McKenna & Williams, 2017). Horizontal PAL in particular allows the individuals involved to establish a way and a pace of learning that is appropriate for them, and enables “meaning-making” to occur ( Havnes, 2008). This process is likely to yield improved long term and deep understanding of the topic being studied.

Veterinary graduates need more than personal learning skills. They also need to develop the skills to help others learn, including their peers. Indeed, the ability to work with peers is desirable for most careers ( Sampson *et al*., 1999), and the UK General Medical Council specifically requires that doctors should be able to teach others ( GMC, 2009). Consider a veterinary surgeon in general practice. In the course of a day, they may have to help owners understand disease and preventative medicine, teach them to administer treatments, support colleagues performing procedures for the first time, guide veterinary students observing practice and perhaps run tutorials for student nurses. The enhanced “meta-adaptive”, or learning-to-learn skills students gain during true engagement with PAL activities ( Lizzio and Wilson, 2004) are invaluable in developing the ability to help other learn.

The potential learning gains for veterinary students from PAL are clear. Most curricula contain some PAL elements, and will have experienced both successes and failures in their implementation. A very dense timetable means that PAL cannot readily be added as an optional extra; instead it must take the place of other teaching methods and hence there is considerable pressure for this learning activity to be successful. The following describes some of our own experiences on the BVSc.

## Peer-assisted learning in the BVSc

There are nine different formal PAL activities in years 1-3 of the curriculum, of which eight are directly timetabled and occur consistently throughout the year. Four of these activities are described in more detail below:

### Anatomy demonstrators

This vertical PAL initiative gives resitting students an opportunity to demonstrate material in half-year (80 students) anatomy practical classes to students in the year below. Unlike most of our other PAL activities, this was explicitly introduced to support the peer tutors rather than focusing on learning for the peer tutees. The classes occur fortnightly through most of the academic year and are run by a lecturer with students following their directions in small groups with a specimen. Resitting students take on the role of demonstrators and should be the first port of call if students need help. Demonstrators have already experienced the class they are assisting, and passed the associated exam, so preparation is based on revision of previous knowledge and a briefing from staff on how to interact with the class. Resitting students are paid for their time, hence are expected to take responsibility and turn up ‘ready to teach’. The student demonstrators are introduced to the class without any reference to their resit status, simply as demonstrators, and are treated as far as possible on an equal footing with the academic staff present.

### OSPE practice in dissections

This horizontal PAL exercise was introduced to allow students to practice “OSPEs” - a practical *viva voce-*type assessment in all three preclinical years which requires knowledge, manual ability and good communication and discussion skills. Dissection classes occur fortnightly through most of the academic year. To close a dissection class, questions relating to the material are provided to students who test each other on the relevant material. Each student is provided with a question and a model answer to allow them to act as ‘examiner’ and give feedback to the examinee. This is usually done in groups of 4-6 students, and rotated so each student gets a chance to be examiner and examinee. The remaining students provide the examinee with encouragement and pick up on points they may have missed.

### Clinical skills lab

Students are introduced to eight basic manual clinical skills tasks in first year, and a further eight in second year. Third year students practice all 16 skills, and begin applying them to simple clinical scenarios. These tasks, which include blood sampling, cytology, dental prophylaxis and CPCR, are introduced by lecturers, and then demonstrated to small groups in the clinical skills lab. Students then practice the skills, unsupervised, using a horizontal PAL approach for two hours per week throughout the year. Each student is allocated a task in which they receive additional training; they are then responsible for acting as an ‘expert’ in this skill and assisting other students who may need additional help. All students have an ‘expert’ role and all skills are covered within the group - meaning theoretically students should be able to access help with any of the skills within their immediate peer group. Online written, audio and video resources are provided, and staff are present for troubleshooting at least twice per term for each group as well as via an online discussion forum.

### Clinical skills troubleshooting clinics

This student-mediated vertical peer learning exercise was implemented to provide additional support to students struggling with clinical skills tasks. Third year students, who have already passed two clinical skills assessments, offer bookable troubleshooting sessions for one afternoon per week throughout the academic year. Attendees stipulate which skills they wish to address when booking in, enabling the peer tutors to arrange their time appropriately. This class is entirely student-organised and run, with booking occurring through a social media platform.

## Success and failure

The peer learning activities described above were approached by the same group of teachers with the same good intentions and equal care in construction, yet there have been significant differences in student perception of, engagement with, and hence the “success” of these sessions. Success in this instance is determined by student feedback consensus, attendance at classes and academic gains in the subject being studied. These two case studies serve to illustrate an apparent success and an apparent failure from our experiences:

### Anatomy demonstrators - a success story

This activity was piloted with one student who had a particular issue with confidence affecting their ability to perform well in practical oral examinations, and was so dramatically successful that it was, last year, rolled out to all resitting students.

It is optional, but resitting students are strongly advised to participate, and the great majority do so. Thus far the feedback is overwhelmingly positive from both the resitting student demonstrators and the learners in class. Demonstrators value the opportunity to revise key subject matter and gain confidence from being trusted by academic staff despite their resit status. Recipients find the student demonstrators approachable and knowledgeable, and staff value the additional support in these intense classes.

Feedback aside, demonstrators who truly engaged with the process show a real and sustained improvement in examination performance and confidence, not just in anatomy but across the board. In addition, participation in this activity improved resitter engagement with the school, and helped keep track of these students who, having left their original cohort, can easily feel isolated and disenfranchised.

### Clinical skills near-peer learning - a mixed bag

This teaching approach was implemented to encourage deliberate practice in the development of these skills ( Ericsson, 2008) and to inspire students to take responsibility for their own learning and truly understand the skills under development, rather than simply imitate the instructor.

Initially, students were instructed to work in pairs using resources provided to develop the manual skills in question. Staff input was minimal and only in the form of extra, bookable, troubleshooting sessions. Student feedback demonstrated that students felt adrift and rapidly lost motivation without a staff presence, and attendance in the lab became noticeably poor. We introduced three main interventions in the last two years, to endeavour to improve this:

• Introduction of a reflective diary, available online, as an app and in paper format, enabling students to easily track their progress and plan learning
• Creation of a student “expert” role, giving every student additional training and responsibility for one skill to enable them to better assist their peers and to try to increase their sense of ownership of the lab. Experts were taught the skill by staff, then performed a supervised demonstration to their peers to initiate the “see one, do one, teach one” learning cycle. Thereafter students were expected to learn skills as before, with the additional support of having a more experienced peer to turn to.
• Increased staff presence in the lab, with five additional staffed “troubleshooting” sessions per student group per year.

Students who engaged with the diary and expert role found their experience improved and their learning and motivation in the lab increased. The majority, however, did not engage well, and continued to struggle. The expert role led some students to become over-burdened with demands of their classmates, and patchy attendance meant that frequently the experts for certain skills were not present. The increased staff presence was perhaps the most counterproductive intervention, and simply resulted in students demanding ever more support and, rather than the intended troubleshooting, the staffed sessions became direct teaching.

## Discussion

The two examples above illustrate that, while PAL can be a successful learning approach, it is not easy to implement and is not guaranteed to be a success. Capstick (2003) reminds educators not to be downhearted about apparent failures - these are common to most teachers trying to implement PAL for the first time. What we, as educators, can do is to reflect on successes and failures and ask ourselves the question: “Why did one work well and the other, not?”

So why did the anatomy demonstrators initiative go so well and the clinical skills lab system struggle so much? Considerably more time, effort and thought went into the clinical skills, in fact, and it is more pressing for this to be a success given PAL is the main learning approach used for this subject in the BVSc. There are numerous contributing factors to the discrepancy between these activities, and neither is perfect.

Our first mistake, and one which seems to trip educators up again and again, is that we were guilty of assuming students are already mature learners, when in fact most of them are fresh from school and unused to taking responsibility for their learning. In a heavy course, the default setting of our students is often to want direct teacher contact, believing, from prior experience, this to be the most efficient way of learning. The clinical skills staffed sessions suffered acutely from this; the constant refrain from students was “Can you just go through the whole thing with me?”, particularly as the end of term and summative examinations loomed.

Added to their preconceptions of what constitutes effective teaching, particularly with increased cost of study, is the possibility of students viewing themselves as consumers rather than colleagues in learning - the authors have had direct comments from students along the line: “I’m paying you to make sure I know this stuff”.

Veterinary and medical students are, in general, high achieving and driven and often suffer badly from culture shock when they discover everyone else is equally clever, and being top-of-the-class is no longer a given ( Zenner *et al*., 2005). They tend to be heavily assessment focussed, always fixated on the perceived “end goal” of graduating and entering the profession, and asking them to learn with and from their peers often leads to worry about the validity of the learning this creates ( Glynn *et al*., 2006) - a common theme is: “how do I know if this is exactly what will be in the exam unless I ask a member of staff?”. Of course, aligning assessment to ensure we are assessing the right things in the right way is a whole different topic - if we get that right, students won’t be led to ask that question. Most students seem to give little consideration to learning after graduation, and this presents us with a further challenge - helping students identify and understand their academic responsibilities throughout their careers.

Given their desire for staff contact, it is inherently difficult to “sell” peer learning activities to students. Brookfield (2009) agrees that students need to believe that self-directed learning is conducive to success - teaching staff need to demonstrate the benefits for students to embrace self- and peer-assisted learning. It should be a simple enough matter, in theory. Any student could bring to mind numerous occasions in their non-academic life, when they have had to work out how to do something from first principles, alone or with friends, and most would agree that this led to better learning than if they had had their hand held throughout. The problem appears to lie in making the link between this type of learning and the learning they do in academia. Many students seem to have put their learning in boxes - *this* type for normal life, *this* type for school. The success of the anatomy demonstrating partially lies in the fact that we can demonstrate success in context, and students see the benefits immediately.

The matter of PR is a key difference between these two activities. The anatomy demonstrating is optional, and “sold” to the students as having yielded great success in the past. The resitters are approached as a group, by a staff member they know and trust, and advised as to how this activity would benefit them specifically. At the beginning of the resit year, these students are often anxious and unsure of their position in the school, and offering them this opportunity makes them feel important, useful and wanted. Conversely, the clinical skills PAL is introduced in a relatively informal session during week one of first year, (which also happens to be “Vet Freshers’ Week”, when academic matters are not necessarily at the forefront of most student minds) and students are simply told how it will be with little context given.

Another obvious difference is that one activity is horizontal, with students in the same cohort working together, and the other is vertical, with more experienced students adopting the role of peer tutor. The difference between vertical and horizontal peer learning is worthy of further exploration, in that the aim of each is slightly different. The very nature of vertical peer learning relies on a more experienced student leading less experienced, and there is clear evidence that this enables learning gains due to the “safe space” it creates for less confident students to ask and answer questions of a less intimidating student guide rather than a lecturer ( Glynn *et al*., 2006). We must be cautious, however, of the danger that this simply becomes a different version of passive learning for the peer tutees ( Ladyshewsky, 2000), with peer tutors simply feeding them information. If this is what students are expecting, then no wonder that they struggle with the horizontal peer learning where nobody is particularly experienced.

A mistake we made was in the naming of student “experts” in the clinical skills lab. This changed the student perception from the intended one of collaborative, horizontal peer learning to a more pseudo-vertical teacher-pupil approach, with the added issue that the “experts” were inadequately expert for this to work. A common negative of peer learning, particularly with horizontal approaches, is lack of confidence in the peer tutor ( Williams & Reddy, 2016) and this certainly manifested in the clinical skills lab. We fell prey to giving students what they said they wanted (someone to teach them), rather than focussing on what they actually needed (confidence in their own learning abilities), landing up in a halfway house that worked for relatively few. Terminology matters here. The use of the terms “tutor”, “expert” and even “consultant” as used by Lizzio and Wilson (2004), have instructive, rather than collaborative overtones. This is, of course, fine if that is the intention of the activity, but in the clinical skills lab we want to foster a collaborative approach to learning and undermined that by injudicious choice of terminology.

Giving responsibility is another key, and related, point, and one which we dealt with well in one activity and poorly in another. In the anatomy demonstrator activity, participants are introduced to the class as demonstrators (not resitting students) and the class is instructed to treat them as staff. This has the dual benefit of giving a confidence boost to the demonstrators, and giving them responsibility for their peers’ learning. The demonstrators are warned in advance of this, and told to arrive at the class prepared to teach. Additionally, there are only two demonstrators in each class of around 80 students - there is nowhere to hide. The perceived potential for exposure and discomfort in the class, although the demonstrators are in truth heavily backed up by staff, act as an effective stimulus to ensure students prepare well, and these students truly engaged with the process. Lizzio and Wilson ( Lizzio & Wilson, 2004) demonstrated that students engaged in their vertical peer “consultant” system show improved learning approaches and meta-adaptive skills, which certainly appears to be reflected in the experiences of our anatomy demonstrators. In contrast, the clinical skills “expert” role failed partly because of the lack of monitoring and the relative anonymity of the role, partly due to inadequate preparation and partly because there is no immediate perceived consequence of poor performance - students felt no real responsibility. In each group of twenty-eight students, there are three or four experts for each skill and it was clear that this resulted in the over-loading of some students, while others shirked their responsibilities entirely.

Attendance is a factor which commonly impacts upon PAL schemes ( Capstick, 2003), and the absence of staff from most clinical skills sessions made it easy for students to simply avoid engagement with this learning approach. Conversely, the anatomy practicals are staffed and compulsory for the students in the class, hence students have little choice but to engage. The anatomy practicals are routinely rated as the most useful learning activity by students, and it is interesting to note that their positive experiences in learning in anatomy practicals, using the practice OSPEs and learning with the anatomy demonstrators, do not seem to be used to improve their approaches to learning in other subject areas. Perhaps another reminder that these are relatively inexperienced learners, for all their academic achievement prior to university.

Both the activities under discussion have been implemented by staff - they are teacher-mediated peer learning activities ( Havnes, 2008). Motivation is a major factor as discussed above, and from this it is simple to extrapolate that student led, peer-mediated, activities may lead to greater engagement and success. This is an avenue which educators would be well advised to exploit, and which has led to some success in our own institute - it is particularly interesting that one of our most well-received forms of PAL is the clinical skills student-led troubleshooting clinic, coordinated and delivered by our 3 ^rd^ year students. This was in fact instigated by one of the authors, but has been advertised, designed and organised by the students themselves and is regularly named in surveys as one of the most useful forms of feedback the students receive. Students involved in in this activity are self-selecting and hence predisposed to engagement - this is a drawback, since it is likely that some of the students most in need are not participating, and warrants further development of this successful activity.

For peer learning to be effective, there needs to be very clear guidance in terms of aims and learning objectives, and student preparation for handling subject matter and approach to learning ( Sampson *et al*., 1999). It is this last factor that is perhaps the most crucial. As experienced teachers, it is easy to forget our initial struggles in helping others to learn effectively; in designing a course, managing time, providing opportunities for exploration and discussion and in catering for a diverse learning population. In overlooking these factors when preparing students to learn in a PAL environment, we are setting them up to fail. Our anatomy demonstrators are at ease with this aspect of their role - the class is still run by a staff member and follows a well-established format; the demonstrators are clearly briefed as to their role and how best to help the other students, and armed with tools to keep students learning actively (e.g. questions to ask etc). Our clinical skills horizontal PAL lacks this level of preparation. Students are told that they are “doing deliberate practice”, but no time is dedicated to helping them learn to learn in this way, and in truth we were somewhat guilty, as Topping and Francis (2005) put it, of “putting children together and hoping for the best”.

Resources may play a part in influencing engagement with peer learning activities (indeed, all learning activities). At a recent workshop run by the authors at an education conference, we observed unexpected differences in engagement within peer learning within the same group depending on the resources they had been given - video versus written instructions. In the anatomy practical classes, students only have access to the dissection guides and each other. They are not permitted any devices or notes in the class, and hence there are minimal distractions from the tasks at hand and a collegiate atmosphere is fostered. In the clinical skills lab, however, many provided resources are online and in video format, which tends to lead to students sitting watching the videos in silence, rather than problem-solving together, and provides temptation to access other, unrelated online forums and social media platforms which distract students from their studies and wastes time.

Lastly, but crucially, the role of formative assessment and feedback must play a vital part in successful self-directed or peer-assisted learning. We may wish it to be otherwise, but it is known that assessment drives learning (Chana, 2008) and we discussed above how assessment-focussed these students tend to be. The main driver for our students tends to be the accumulation of credit towards progress, hence formative assessment is possibly not a motivator in its own right. Nonetheless, well-constructed, timely, relevant formative assessment enables students to track their learning and prepare for summative assessments as well as providing useful feedback and encouraging self-audit and mature learning approaches (Earl, 2003; Boud and Falchikov, 2005).

The difference in our two case studies is clear again here. The anatomy demonstrators are themselves engaging in a form of formative assessment by answering student queries and leading discussions around the questions provided in dissection guides, and the students in the class also receive highly specific formative assessment via these same questions and through the OSPE practice which closes each session. Both demonstrators and students therefore leave the class having learned and tested their knowledge, which enables them to go forward confidently to address any knowledge gaps.

In contrast, there is no continuous form of formative assessment built in to the clinical skills lab. Students are expected to receive feedback through the deliberate practice process ( Ericsson, 2008) but, as discussed, this process has not been engaged sufficiently to effect this benefit. Students do undertake a mid-year formal formative assessment in all subjects, including clinical skills, presented in the same format as the summative examinations. Students greatly value this opportunity, but it probably comes too late and occurs only once, plus feedback is delivered at least four weeks after the event and is too generic in many cases to be truly useful. This lack of ongoing assessment and feedback in the lab contributes to the lack of confidence in the PAL system and reinforces the suspicion, possessed by many students, that their peers may lead them to learn things incorrectly. A potentially powerful tool to consider may be building PAL in the clinical skills lab into a cumulative form of summative assessment, simultaneously emphasising the relevance of gaining learning skills and providing both feedback and that ever-desirable exam credit ( Boud *et al.*, 1999).

## Summary and future plans

Reflecting on our peer learning activities to date has brought into relief the reasons for success of some and failure of others. Key focuses for success seem to include:

• PR and proof of principle - students need to believe in the process
• Sufficient guidance and support from staff in the process of learning in this way
• Appropriate use of vertical vs horizontal PAL, depending on the purpose of the activity
• Careful consideration of resources used and the effect these may have on the class dynamic
• Formative assessment

Some authors, including Capstick (2003) and Topping and Francis (2005) have provided useful guides to help educators design fruitful PAL activities, and there is much sympathy and support in the literature for teachers who have struggled to implement these successfully - failure is common, even for those who have used PAL successfully before, and does not mean PAL is inappropriate in itself. Our own experiences echo this and remind us that a reflective approach to teaching is crucial if one is to avoid throwing the baby out with the bath water, when an activity does not answer as expected.

To address our shortcomings, we are implementing a number of changes based on our reflections above, including:

• Introduction of collaborative-learning workshops in our study skills stream, using a fun and challenging task (e.g. origami) to replicate the informal PAL students undertake unconsciously. A discussion following this will aim to stimulate the realisation that they already have the skills they need to engage successfully with PAL in more formal settings.
• Recruiting and training peer academic supporters, to work alongside our excellent pastoral peer supporters
• Clinical skills enhancements
  • Providing formative assessments regularly in the clinical skills lab, and working towards a continuous assessment model
  • Changing the emphasis of the “expert” role to a buddy system
  • Making the reflective diary part of the assessed portfolio to increase usage
  • Removing staffed troubleshooting sessions in favour of promoting the student-led vertical PAL sessions

We also plan to build on our successes; for example we will be consulting students to identify appropriate areas to introduce additional student-mediated vertical PAL sessions for key difficulties - one suggestion was a numeracy support group for students struggling with clinical calculations.

The remaining itch at the back of our necks is that query touched on above - why is it that students appear to exhibit good learning approaches in one class but fail to transpose those skills to other subjects and learning environments? We will be undertaking a research project evaluating student engagement with self-directed and peer-assisted learning activities, and the effect on learning skills, in the hope of going some way to answering this.

## Conclusion

Peer-assisted learning, whether student or teacher-mediated, is becoming an essential tool for today’s veterinary educators. The devil is in the detail, as they say, and any teacher considering increasing student-led or peer-assisted learning in their teaching would be well advised to consider the lessons we have learned:

• Get students on board from the start
• Design the activity to suit its purpose, not reduce teaching staff input
• Provide sufficient guidance in learning and teaching methods for the students involved
• Provide plentiful formative assessment opportunities
• Use resources appropriately and with care
• If it’s not working, ask why not!

## Notes On Contributors

Alison Reid BVMS PGCertHE FHEA MCRVS is a lecturer in veterinary preclinical sciences and clinical skills at the University of Liverpool, UK. Alison leads the Institute educational research group, and is the academic lead for year 2 of the BVSc curriculum.

Rosie MacDiarmid BSc (Hons) BVM&S FHEA MRCVS is a lecturer in veterinary preclinical sciences and clinical skills at the University of Liverpool, UK. Rosie leads the teaching of exotic species’ anatomy and physiology in the BVSc.

Emma Ormandy BVMS (Hons) MSc MRCVS is a lecturer in veterinary professional and clinical skills at the University of Liverpool, UK. Emma is the academic lead for teaching of professional skills in the BVSc.

Karen Noble BVM&S DBR PGCertHE FHEA PhD MRCVS is a senior lecturer in veterinary preclinical sciences at the University of Liverpool, UK. Karen is the academic lead for year 1 of the BVSc, and is the director of preclinical extramural studies.

Fay Penrose BA (Hons) PGCertHE FHEA is a lecturer in veterinary preclinical sciences at the University of Liverpool, UK. Fay is the academic lead for normal structure and function in the BVSc, and leads outcomes assessment within the Institute.
