# Supplementary material for: Peer-assisted learning - an antidote for spoonfeeding? Reflections on peer-assisted learning activites in a veterinary curriculum
Source: MedEdPublish (2016). 2017 Sep 15;6:159. [Version 1] doi: 10.15694/mep.2017.000159 (PMC10885292; doi:10.15694/mep.2017.000159)
Supplement: Peer review history

# Reviewer report: Reviewer response for version 1

**DOI:** 10.21956/mep.19347.r28278

**Published:** 2018-09-29

**Authors:** Anthony David.M

**Affiliations:** 1 Soho

This review has been migrated. The reviewer awarded 4 stars out of 5 A good work presented comprehensively. Though it is a long read, it is worth. The comparison of a successful and an apparently less successful attempt and elucidating the reasons is very thought provoking. The fact that assessment drives learning seems to be a universal adage in medical and even other professional education. The density of the curricular program and the type of students described makes it easier for others to try out PAL in similar scenarios.

Reviewer Expertise:

NA

No decision status is available

# Reviewer report: Reviewer response for version 1

**DOI:** 10.21956/mep.19347.r28276

**Published:** 2017-10-05

**Authors:** Julie Hunt

**Affiliations:** 1 Lincoln Memorial University

This review has been migrated. The reviewer awarded 4 stars out of 5 This article compares the apparent success of a PAL program in anatomy training with the seemingly lesser degree of success experienced by a PAL program in clinical skills training at the same veterinary institution. I appreciate the authors' willingness to share their description and interpretation of the clinical skills PAL program despite its lack of success per the authors. Understanding what doesn't work and why is at least as helpful as understanding what does work and why. In this case, the authors do a good job of comparing and contrasting the two programs and making some meaning out of why one was more successful than the other. This article would be helpful reading for educators looking to start a PAL program.

Reviewer Expertise:

NA

No decision status is available

# Reviewer report: Reviewer response for version 1

**DOI:** 10.21956/mep.19347.r28277

**Published:** 2017-09-25

**Authors:** John Dent

**Affiliations:** 1 AMEE

This review has been migrated. The reviewer awarded 4 stars out of 5 This paper provides a thoughtful overview of the experiences gained from implementing a PAL programme for veterinary studentsIt looks particularly at the results experienced in two different scenarios and comments on possible reasons of the success and difficulties encountered. The paper provides information of interest to anyone considering the implementation of a similar programme but I think could be made clearer if it was presented in the 12 tips format popular in Medical Teacher.

Reviewer Expertise:

NA

No decision status is available
